# Curcumin attenuates combined DON+AFB1 induced apoptosis via Nrf2-associated signaling in porcine alveolar macrophages 3D4/21 cell line: an *in vitro* study

**DOI:** 10.3389/fvets.2026.1949221

**Published:** 2026-10-28

**Authors:** Azhar Muhmood, Han Guocong, Mostafa A. Abdel-Maksoud, Abdulaziz Alamri, Saeedah Almutairi, Kehe Huang

**Affiliations:** 1 Key Laboratory of Tropical Biological Resources of Ministry of Education, School of Pharmaceutical Sciences, Hainan University, Haikou, China; 2 Research Chair of Biomedical Applications of Nanomaterials, Biochemistry Department, College of Science, King Saud University, Riyadh, Saudi Arabia; 3 Biochemistry Department, College of Science, King Saud University, Riyadh, Saudi Arabia; 4 Botany and Microbiology Department, College of Science, King Saud University, Riyadh, Saudi Arabia; 5 College of Veterinary Medicine, Nanjing Agricultural University, Nanjing, Jiangsu, China

**Keywords:** antioxidants, apoptosis, curcumin, mycotoxins, Nrf2/HO-1 signaling

## Abstract

Deoxynivalenol (DON) and Aflatoxin B1 (AFB1) are among the most prevalent mycotoxin contaminants of grain based feed and frequently co-occur, yet most evidence on their combined toxicity comes from hepatic, intestinal, or lymphoid models. Alveolar macrophages are among the first immune cells to encounter mycotoxins carried on inhaled feed dust in swine housing, this respiratory exposure route is toxicologically distinct from oral uptake and has not been characterized DON+AFB1 combination. In the present *in vitro* study, the porcine alveolar macrophages (PAMs) cell line 3D4/21 was chosen as a model and treated by various combined concentrations of DON+AFB1. Co-exposure induced cytotoxicity in a dose-dependent manner, as evidenced by reduced cell viability, elevated LDH release, a suppressed Bcl-2/Bax ratio, and increased intracellular ROS accumulation. These changes are consistent with activation of the intrinsic apoptotic pathway. Curcumin, a natural product widely used in traditional Chinese medicine and known for its antioxidant properties, increased cell viability, reduced LDH release and ROS accumulation, and reversed the apoptotic shift in Bcl-2/Bax/Cytochrome c and Caspase-3 expression. DON+AFB1 co-exposure also suppressed the Nrf2/HO-1/NQO1 antioxidant axis, whereas curcumin restored Nrf2, HO-1, and NQO1 expression at both mRNA and protein levels. Pharmacological inhibition of Nrf2 with ML385 reduced the protective effects of curcumin by preventing restoration of the antioxidant gene expression and elevating apoptotic markers. These findings indicate that Nrf2/HO-1 signaling is associated with the protective effects of curcumin against DON+AFB1 induced oxidative stress and apoptotic changes in PAMs and support further evaluation of curcumin as a candidate intervention against inhalation mycotoxin co-exposure in swine production environments.

## Graphical Abstract

**Figure:**
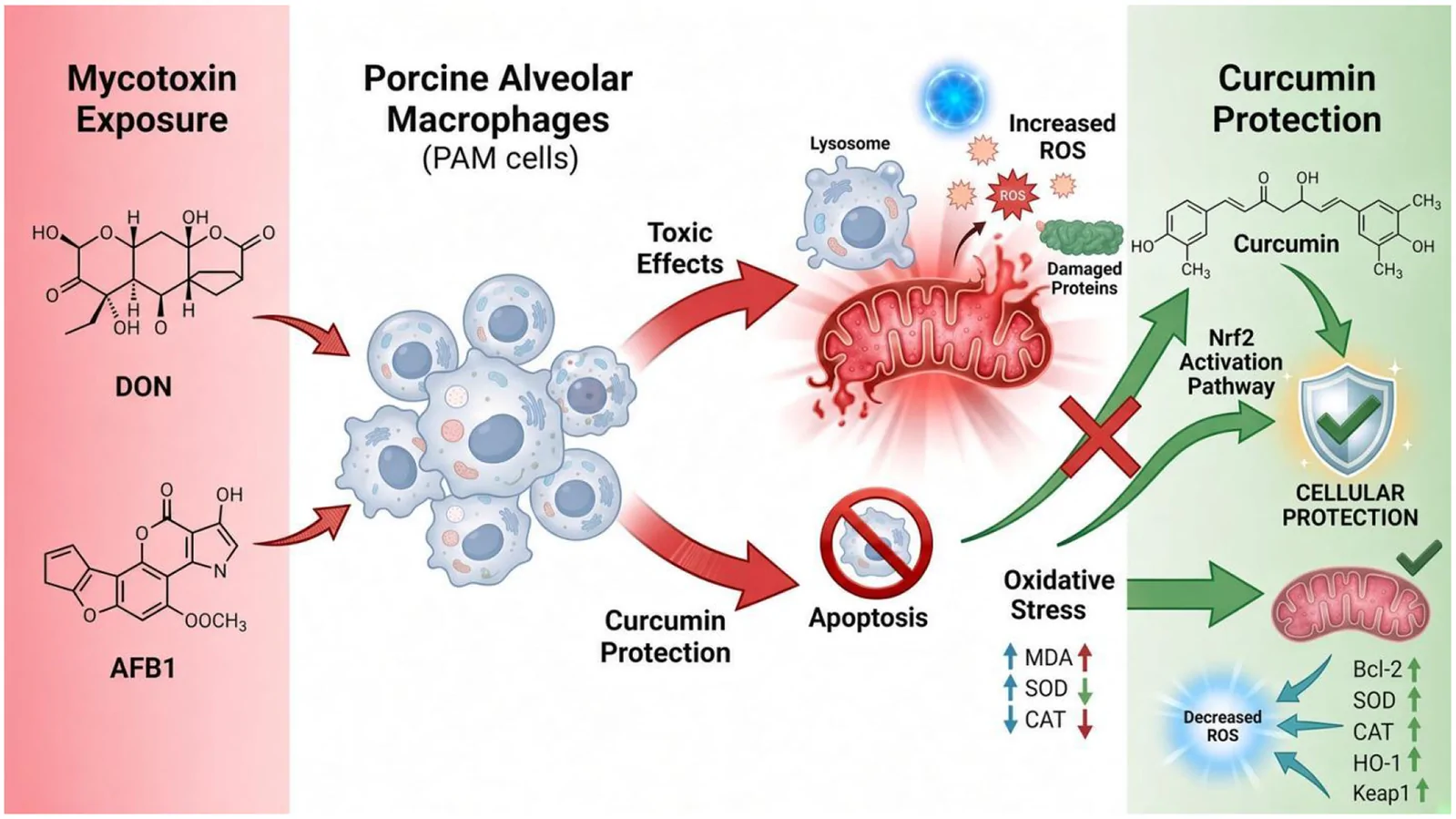

## 1 Introduction

Mycotoxin is specified as a chemically separate category of secondary fungal metabolites yield by the *Aspergillus, Penicillium*, or *Fusarium* genus and their presence widely reported in the environment (1). The FAO has estimated that 25% of the world food supply is contaminated with these mycotoxins (2). Deoxynivalenol (DON) and aflatoxin B1 (AFB1) are among the most common and unavoidable mycotoxin contaminants of grains, feed, and food worldwide, and both pose serious risk to human and animal health (3). DON is a common trichothecene mainly produced by Fusarium species, contaminates wheat, maize, and barley, disrupts protein synthesis by binding the 60S ribosomal subunit, producing ribotoxic stress, immunosuppression, and growth impairment across species (4). It also induces apoptosis and oxidative stress/mitochondrial dysfunction in epithelial and immune cells (5). AFB1, produced by *Aspergillus flavus* and *A. parasiticus*, classified by the IARC as Group 1 hepatocarcinogen, is metabolically activated to AFB1-8,9-epoxide, forming DNA adducts, inhibiting transcription and triggering oxidative damage, as well as apoptosis (6).

Co-contamination of feed with DON and AFB1 is a documented and ongoing concern in the European Union, AFB1 is regulated at a maximum of 0.02 mg/kg in complete pig feed (*Directive 2002/32/EC*), and DON at a guidance value of 0.9 mg/kg feed for pigs (*Commission Recommendation 2006/576/EC*), yet a recent EU feed monitoring survey using validated LC-MS/MS methods detected co-occurring AFB1 at 0.003–0.042 mg/kg and DON at 0.16–0.95 mg/kg across sampled feed, confirming that the real world contamination levels frequently approach or exceed current regulatory limits (7). DON and AFB1 frequently co-occur in the same feed and grain sources, and their co exposure has been shown amplifying oxidative stress, mitochondrial membrane permeabilization and caspase dependent apoptosis across the liver, spleen, and intestine with corresponding disruption of the Bax/Bcl-2 balance and cytochrome-c (8). These effects extend beyond livestock productivity to human health, where dietary exposure has been linked to stunted growth, immune dysfunction, and increased infection susceptibility in vulnerable populations (9).

Mycotoxins reach the respiratory tract through inhalation of aerosolized dust generated from handling, milling, or storage of contaminated grain and bedding material in swine housing, a well-documented occupational and animal health exposure route that is mechanistically and kinetically distinct from oral or systemic uptake (10). Dust samples collected from grain processing facilities and animal housing environments, including cowsheds and poultry sheds have been found to contain aflatoxin, ochratoxin A, and zearalenone, highlighting these environments as genuine sources of occupational exposure (11). Following inhalation, mycotoxins act directly on the respiratory epithelium. Aflatoxin reduces ciliary beat frequency, compromising mucociliary clearance and thereby weakening protection of the lower airways against pathogens and particulates. Repeated AFB1 exposure *in vivo* produces alveolar epithelial hyperplasia, interstitial and vascular inflammation, macrophage infiltration, as well as increased lung tissue apoptosis (12). Occupational and agricultural inhalational exposure has been associated with cough, wheeze, other respiratory complaints, and higher pulmonary cancer risk in highly exposed population (13). It remains unclear whether oxidative injury and intrinsic apoptosis induced by combined DON and AFB1 also occur in porcine alveolar macrophages.

Natural compounds with antioxidant activity have been examined as possible countermeasures. Curcumin, the principal polyphenol of *Curcuma longa*, has well-established antioxidants, anti-inflammatory and anti-cancer activity (14), and can activate nuclear factor erythroid 2-related factor 2 (Nrf2), which induces heme oxygenase-1 (HO-1) and other antioxidant enzymes. Curcumin has been reported to limit oxidative injury from other mycotoxins, including zearalenone-induced apoptosis in Leydig cells via a PTEN/Nrf2/Bip axis (15).

It is unknown whether the protective effect of curcumin is truly associated with Nrf2 activation, or Nrf2 activation is only an accompanying event. Prior studies have shown Nrf2/HO-1 pathway components changing alongside protective effects of curcumin (16) but few have used pharmacological Nrf2 blockade in a mycotoxin co-exposure model.

The present work is an acute *in vitro* study in a single porcine alveolar macrophage line (3D4/21). Combined DON+AFB1 was applied in culture medium to characterize cytotoxicity, oxidative stress and intrinsic apoptosis, and to test whether curcumin attenuates these responses. The selective Nrf2 inhibitor ML385 was then used to test whether that attenuation is associated with Nrf2 signaling. The study was not designed to quantify inhaled dose, plasma concentration or *in vivo* respiratory protection.

## 2 Materials and methods

### 2.1 Cell line

The PAM 3D4/21 cells line were purchased from the China Institute of Veterinary Drug Control (Beijing, China) and preserved in our laboratory at passages 15–20. Cell identity was confirmed by the supplier documentation and by typical PAM morphology under phase contrast microscopy. Cultures were routinely examined and confirmed free of contamination before experimental use. Cells were cultured in DMEM medium (Catalog No. CM15019, Macgene, Biotech, Beijing, China) in the presence of 10% fetal bovine serum (Catalog No. ST30-3302, FBS, PAN-Biotech, Germany) and 1% antibiotics (100 U/mL penicillin and 100 μg/mL streptomycin Gibco, USA) at 37 °C, with 5% CO_2_ in a humified incubator (Thermo Fisher Scientific, USA).

### 2.2 Chemical and reagents

Aflatoxin B1 and Deoxynivalenol (AFB1 and DON; purity ≥99.8%) was purchased from Qingdao Pribolab Biological Engineering Co., Ltd. (Qingdao, Shandong, China) Curcumin was obtained as a yellow crystalline powder from (Sigma Aldrich, St. Louis, MO, USA; catalog no. 08511; MW 368.38 g/mol; purity ≥98.0%), Radio immunoprecipitation assay lysis buffer (RIPA) and BCA analysis kit (P0013B, P0012S, Beyotime, China), 3-(4,5-dimethyle-2-thiazolyl)-2, 5-diphenyl-2-H-tetrazolium bromide (MTT) and horseradish peroxide (HRP)-conjugated goat anti-rabbit secondary antibodies (7076) were purchased from Sigma-Aldrich (St. Louis, MO, USA). Primary antibodies caspase-3 (CST, 9662, 1:1,000), Bcl-2 (Wanleibio, China, 1:1,000), Bax (Abclonal, China, 1:1,000), Cyt-c (Wanleibio, China, 1:1,000), Nrf2 (Beyotime, China, AF7623, 1:1,000), NQO1 (Cell Signaling Technology, C#62262, 1:1,000), HO-1 (Beyotime, China, AF1333, 1:1,000), and GAPDH (ABclonal, AC002, 1:2,000), ECL (Beyotime, China, P0018S). The inhibitor ML385 (Nrf2 inhibitor) was obtained from MedChemExpress (Monmouth Junction, NJ, USA), dimethyl sulfoxide (DMSO) was purchased from Beyotime, China (ST038).

### 2.3 Cells treatment and experimental design

Porcine alveolar macrophage 3D4/21 cells were seeded in 96-well plates at the density of 8 × 10^3^ cells per well and allowed to attach before treatment. Cells were maintained in a humidifier incubator at 37 °C with 5% CO_2_. The culture medium was replaced every 12–24 h according to cell growth status. When cell confluence reached approximately 80%, cells were washed three times with phosphate buffer saline (PBS), detached with trypsin, resuspended, and seeded into 96, 12, or 6 well-plates according to experimental requirements. To evaluate the effect of curcumin on combined DON and AFB1 exposure, four experimental conditions were established: (i) untreated control (PBS containing ≤ 0.1% ethanol), (ii) DON (0.25, 0.5, 1.5, or 2 μg/mL) + AFB1 (0.02, 0.04, 0.08, or 0.16 μg/mL), (iii) curcumin alone (1, 2, 4, or 8 μM), (iv) Combined DON (0.5 μg/mL) + AFB1 (0.04 μg/mL) + curcumin (2 μM). A second set of experiments tested whether NRF2 inhibition altered effect of curcumin. Groups were (i) untreated control (PBS containing ≤ 0.1% ethanol) (ii) DON (0.5 μg/mL) + AFB1 (0.04 μg/mL), (iii) DON (0.5 μg/mL) + AFB1 (0.04 μg/mL) + curcumin (2 μM), (iv) DON + AFB1 + curcumin + ML385 (5 μM). In the inhibitor experiments, cells were pretreated with ML385 for 4 h and then exposed to curcumin and/or the mycotoxin combination for 24 h.

### 2.4 MTT assay

Cell viability was evaluated in 3D4/21 cells exposed to AFB1+DON and curcumin under the experimental conditions described above. 3D4/21 cells were seeded into 96-well plates at the density of 8 × 10^3^ per well allowed to adhere for 24 h, and treated with AFB1 + DON and curcumin, their combination, or vehicle control. At 24 h post exposure, the culture medium was replaced with fresh medium containing 15 μL of MTT reagent (5 mg/mL, Sigma, USA). Plates were incubated at 37 °C for 2–4 h in the dark to allow intracellular formation of formazan crystals. The MTT solution was then removed, and 1 mL dimethyl sulfoxide (DMSO) was added to solubilize the formazan. Plates were gently agitated for approximately 10 min at room temperature until complete dissolution was achieved. A 200 μL aliquot from each well was transferred to a 96-well-plate, and absorbance was measured at 570 nm with a 630 nm reference wavelength using a calibrated microplate reader (Bio Rad USA). Blank absorbance (medium + MTT without cells) was subtracted, and data were expressed as percentage viability relative to the untreated control for each time point (17). All experiments were independently repeated three times to ensure the reliability.

### 2.5 LDH assay

For lactate dehydrogenase (LDH) release, PAM cells were seeded in 96 well-plates at 8 × 10^3^ cells/well, and then subjected to treatment for 24 h. After incubation, the culture medium in each well was gently removed and placed in a microcentrifuge tube. The samples were centrifuged at 12,000 rpm for 15 min at 4 °C to pellet the cells and remove cellular debris. Supernatants were obtained and frozen at −20 °C till further analysis. LDH activity was measured with a commercial LDH assay kit (Jiancheng Bio, A020-2-2, Nanjing, China) as described previously without any modification (18). Each experiment was repeated three times and LDH release is expressed relative to the untreated control group.

### 2.6 Oxidative stress assessment

Oxidative stress was measured in cell lysates as malondialdehyde (MDA) content and as superoxide dismutase (SOD) and catalase (CAT) activities. MDA was determined with a lipid peroxidation kit (Beyotime, S0131S) based on the reaction of MDA with Thio barbituric acid under acidic, high temperature conditions to form a red MDA TBA adduct. Lysates were mixed with the kit TBA working solution, heated according to the manufacturer (15 min at 100 °C), cooled, and the supernatant was read at 532 nm. Concentrations were calculated from an MDA standard curve and reported as nmol/mg protein, following the classic TBA method as previously described (19). SOD activity was measured with a nitroblue tetrazolium kit (Beyotime, S0087/S0109). Superoxide generated by the xanthine/xanthine oxidase system reduces NBT to formazan; SOD in the sample competes for superoxide and limits formazan formation. Lysates were incubated with the NBT working solution and xanthine oxidase reagent as specified by the manufacturer, and absorbance was read at 560 nm. Activity was calculated from the percent inhibition of NBT reduction relative to a blank and expressed as U/mg protein (20). CAT activity was measured with an ammonium molybdate kit (Jiancheng, A007-1-1). CAT in the lysate decomposes a fixed amount of H_2_O_2_ over a set interval; the reaction is then stopped with ammonium molybdate, and residual H_2_O_2_ forms a pale yellow complex that was read at 405 nm. Values were calculated from the kit formula and expressed as U/mg protein (21).

### 2.7 ROS level assay

The PAMs were cultured at a density of 2 × 10^5^ cells/well in six well-plates with corresponding treatment and the intracellular ROS were measured by flow cytometry as described previously with minor modification (22). In brief, after removing the culture medium, cells were washed three times with serum free DMEM and incubated with 10 mM of 20, 7′-dichlorofluorescein diacetates (DCFHDA; Sigma, USA) for 30 min at 37 °C. Subsequently, the cells were washed three times with serum-free DMEM and re-suspended in PBS and the total intracellular fluorescence intensity was measured for more than 10,000 cells of each sample by flow cytometry. The level of total intracellular ROS, paralleled by an increase in fluorescence intensity, was calculated as the percentage of control cells. Laser scanning confocal microscopy was also used to detect the intracellular ROS as described previously with minor modification (23). Unlike the flow cytometry, PAMs were cultured on 20 mm diameter round coverslips (WHB, China) into 12 well culture plates with corresponding treatment. When cells reached 70-80% confluence, they were washed three times with serum free DMEM and loaded with DCFH-DA for 30 min at 37 °C. Finally, cells on coverslips were washed three times with PBS and mounted onto glass slides using Mowiol (Sigma, USA). Samples were photographed using a Zeiss LSM 710 META confocal system with the same scan settings.

### 2.8 Apoptosis analysis

The cell apoptosis was measured by Annexin V-FITC (C1067 Beyotime, China) and propidium iodide (PI) followed by flow cytometry. The PAM cells at 1 × 10^5^ cells/well were cultured in six well-plates. At each exposure time, both floating and adherent cells were collected to avoid loss of apoptotic populations. Adherent cells were detached with 0.25% trypsin EDTA, combined with floating cells, and washed twice with ice cold PBS. Cell pellets were resuspended in Annexin V binding buffer at a density of 1 × 105 cells per 100 μL and stained according to the manufacturer's instructions (BD Pharmingen). Samples were incubated for 15 min at room temperature in the dark, diluted with 400 μL binding buffer, and analyzed immediately (within 1 h) using a BD FACSCalibur flow cytometer at 488 nm. Instrument settings were optimized using unstained, single stained, and fluorescence-minus-one controls. Debris was excluded by forward and side scatter gating, doublets were removed using FSCH vs. FSCA, and a minimum of 10,000 gated single cell events was acquired for each sample. Total apoptosis was calculated by Beckman, coulter, USA, to find the proportion of the apoptotic ells. Flow cytometry data were analyzed using FlowJo v10.

### 2.9 Quantitative real-time PCR

PAMs were cultured at a density of 6 × 10^4^ cells/well in 12 well-plates with corresponding treatment. The total cellular RNA was extracted using the Trizol reagent (TaKaRa, Japan) according to the manufacturer's instructions (24). RNA purity and concentration were determined by NanoDrop prior to reverse transcription. Total RNA (1 μg per sample) was reverse transcribed to cDNA using a reverse transcription kit (Vazyme, China) according to the manufacturer's protocol. Quantitative real time PCR (qRT-PCR) was performed using a CFX384 real time PCR detection system (Bio Rad, Hercules, CA, USA) with SYBR premix Ex Taq (TaKaRa, Japan). Sangon Biotech (Shanghai, China) designed the gene specific primers and Tsingke Biotechnology Co., Ltd. (Wuhan, China) synthesized them. Primer sequences, amplicon sizes, predicted melting temperatures (Tm), and GenBank accession numbers are listed in Table 1. Thermal cycling conditions were as follows: initial denaturation at 95 °C for 10 mins, followed by 40 cycles of denaturation at 95 °C for 15 s and annealing at 60 °C for 1 min. The temperature listed in Table 1 represent the predicted primer melting temperature (Tm) calculated during primer design; a uniform annealing temperature of 60 °C was applied to all primer pairs during thermal cycling, selected to ensure specific amplification across the full gene panel. Relative expression levels of mRNA of target genes were calculated by using 2-ΔΔCt method, with β-actin as an internal reference gene for normalization.

**Table 1:** Primers sequences used for quantitative real-time PCR analysis.

Target genes	Primer sequence (5^′^-3^′^)	Product size (bp)	Melting temperature (Tm)	Accession number
*Nrf2*	F: GCTGCCATTAGTCAGTCGCTCTC	155	67.9 °C	XM_013984303.2
	R: ACCGTGCCTTCAGTGTGCTTC			
*Keap1*	F: TGGGCGTGGCAGTGCTCAAC	158	70.1 °C	NM_001114671.1
	R: GCCCATCGTAGCCTCCTGCG			
*HO-1*	F: GCTCTATCGTGCTCGCATGA	71	65.2 °C	NM_001004027.1
	R: AATTCCCACTGCCACGGTC			
*NQO1*	F: ACATCACAGGGGAGCCGAAGGACT	86	65.3 °C	NM_001159613.1
	R: GGCACCCCAAACCAATACAATG			
*Bcl-2*	F: GGCCTTCTTTGAGTTCGGT	142	63.0 °C	XM_021099593.2
	R: ATACAGCTCCACAAAGGCATC			
*Bax*	F: TGAGCAGATCATGAAGACAGGG	163	63.0 °C	XM_003127290.5
	R: GAGACACTCGCTCAACTTCTT			
*Cytochrome c*	F: GATTCACTTACACAGATGCCA	145	61.2 °C	XM_003125443.6
	R: TTGTTCCAGGGATGTACTTCTT			
*Caspase-3*	F: ACAGCACCTGGTTACTATTC	102	60.0 °C	NM_214162.1
	R: ATTCTACTGCTACCTTTCGG			
*β-actin*	F: GGACTTCGAGCAGGAGATGG	114	65.0 °C	NM_001170517.1
	R: GCACCGTGTTTGCGTAGAGG			

### 2.10 Western blot analysis

Cells were incubated in 6 well plates at the density of 2 × 10^5^ cells/well with corresponding treatment. After washing three times with PBS, the total cellular proteins were extracted with radioimmunoprecipitation assay (RIPA) lysis buffer containing a protease inhibitor cocktail and phenylmethylsulphonyl fluoride (PMSF) (Applygen, Beijing, China) and sonicated (Sonics VCX105) on ice. The cell homogenate was centrifuged at 12,000 rpm for 15 min at 4 °C. The supernatant was collected, and total protein concentration was measured with a bicinchoninic acid (BCA) protein assay kit (Beyotime, China). Forty micrograms of protein samples were denatured in loading buffer and heated at 95 °C for 5 min. protein samples were loaded onto 12% SDS–polyacrylamide gels and transferred to polyvinylidene fluoride (PVDF) membranes. Membranes were blocked with Tris-buffered saline (TBS) containing 5% bovine serum albumin (BSA) for 1 h at room temperature to minimize non-specific binding. The membranes were then incubated overnight at 4 °C with primary antibodies targeting caspase-3, Bcl-2, Bax, cytochrome c (Cyt-c), Nrf2, NQO1, HO-1, and GAPDH. Following primary antibody incubation, the membranes were rinsed with TBST and exposed to horseradish peroxidase (HRP)-linked secondary antibodies (Santa Cruz Biotechnology, CA, USA) for 1 h at room temperature. Immunoreactive protein bands were detected using a BeyoECL Plus chemiluminescent substrate kit (Beyotime, China). These bands were captured with a GE ImageQuant LAS 4000 imaging system (GE Healthcare, USA). Quantitative analysis of band density was performed using ImageJ software (National Institutes of Health, Bethesda, MD, USA).

### 2.11 Statistical analysis

The SPSS software, version 22.0 (IBM Corporation, Armonk, NY, USA), was used for statistical analyses. Results from experiments are presented as mean ± SD of at least three independent replicates. One-way analysis of variance (ANOVA) was used to compare the differences between the experimental groups and the least significant difference (LSD) post hoc test was used to determine the differences between the groups. *P*-value at < 0.05 was assumed to be statistically significant.

## 3 Results

### 3.1 Cytotoxic induced by DON+AFB1 in PAMs

The cytotoxic effect of combined DON+AFB1 exposure was first evaluated in PAMs. Cells were treated for 24 h with paired concentrations of DON at (0.25, 0.5, 1.0, and 2.0 μg/mL) and AFB1 at (0.02, 0.04, 0.08, and 0.16 μg/mL), cell viability and lactate dehydrogenase (LDH) release were measured. Co treatment with DON and AFB1 reduced cell viability in a dose-dependent manner as illustrated in Figure 1A, Exposure to 0.5 μg/mL DON combined with 0.04 μg/mL AFB1 significantly reduced cell viability compared with the control group and was therefore selected for subsequent experiments. Curcumin alone was next evaluated to establish a non-cytotoxic working concentration. Cell viability was significantly reduced at 4 μM and 8 μM curcumin, indicating toxicity at this dose, and concentrations of 2 μM were therefore chosen for further studies (Figure 1B). Curcumin pretreatment improved cell viability to DON+AFB1 toxicity in a dose dependent manner with 2 μM curcumin providing the greatest protection and significantly restored cell viability in combined toxins group, 0.5 μg/mL DON and 0.04 μg/mL AFB1 (Figure 1C). Curcumin alone significantly reduced LDH activity relative to the control group, while DON+AFB1 co-exposure increased LDH release. Curcumin pretreatment mediated this increase, reduced LDH activity toward control levels (Figures 1D, E).

**Figure 1:**
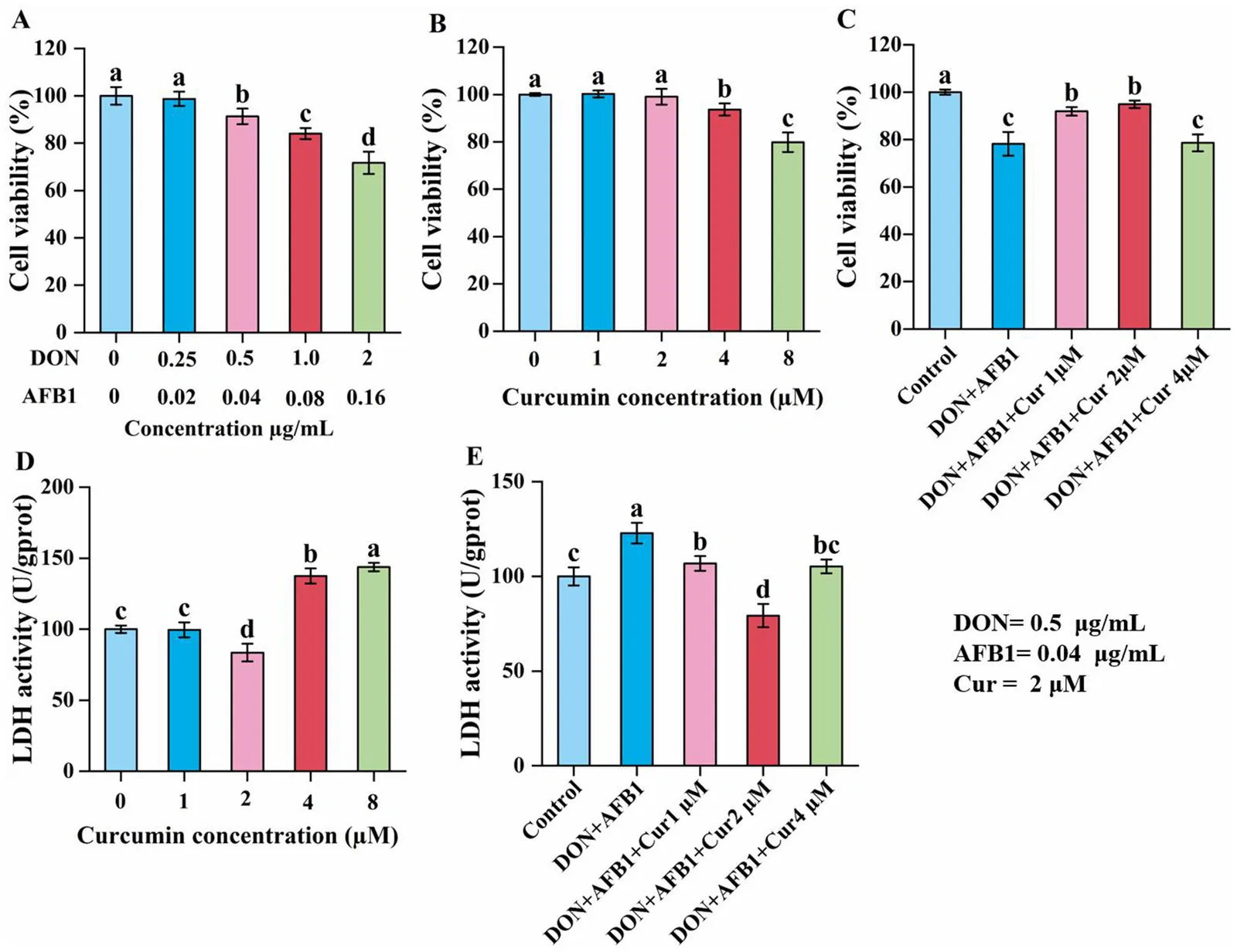
Cytotoxic effects of DON+AFB1 and protective effect of curcumin in PAMs. **(A)** Cell viability following co-exposure to DON and AFB1. **(B)** Cell viability following curcumin treatment alone. **(C)** Cell viability with curcumin pretreatment (1, 2, or 4 μM) prior to DON + AFB1 (0.5 μg/mL + 0.04 μg/mL) co-exposure. **(D)** LDH activity following curcumin treatment alone. **(E)** LDH activity with curcumin pretreatment prior to DON+AFB1 co-exposure. Data are presented as mean ± SD from three independent experiments. Different lowercase letters above the bars indicate statistically significant differences (*P* < 0.05) between groups.

### 3.2 Effect of curcumin on DON+AFB1 induced oxidative stress

To assess the antioxidant effects of curcumin, intracellular ROS, MDA, SOD, and CAT levels were measured in PAMs. The results indicated that, exposure of DON+AFB1 markedly elevated intracellular ROS levels relative to control, and curcumin pretreatment significantly attenuated this increase in ROS production (Figure 2A). Similarly, curcumin pretreatment markedly reduced MDA content that had been elevated by DON+AFB1 exposure (Figure 2B). SOD and CAT activities were significantly reduced by DON+AFB1 treated group relative to control, while curcumin pretreatment restored the content of CAT and SOD relative to combined toxins group (Figures 2C, D). Confocal imaging with a ROS sensitive fluorescent dye showed control and curcumin alone groups showed minimal fluorescence, DON+AFB1 treated cells showed intense fluorescence while curcumin pretreatment markedly reduced this signal (Figure 2E). Together, these results indicated that curcumin associated redox homeostasis in PAMs against oxidative stress induced by DON+AFB1.

**Figure 2:**
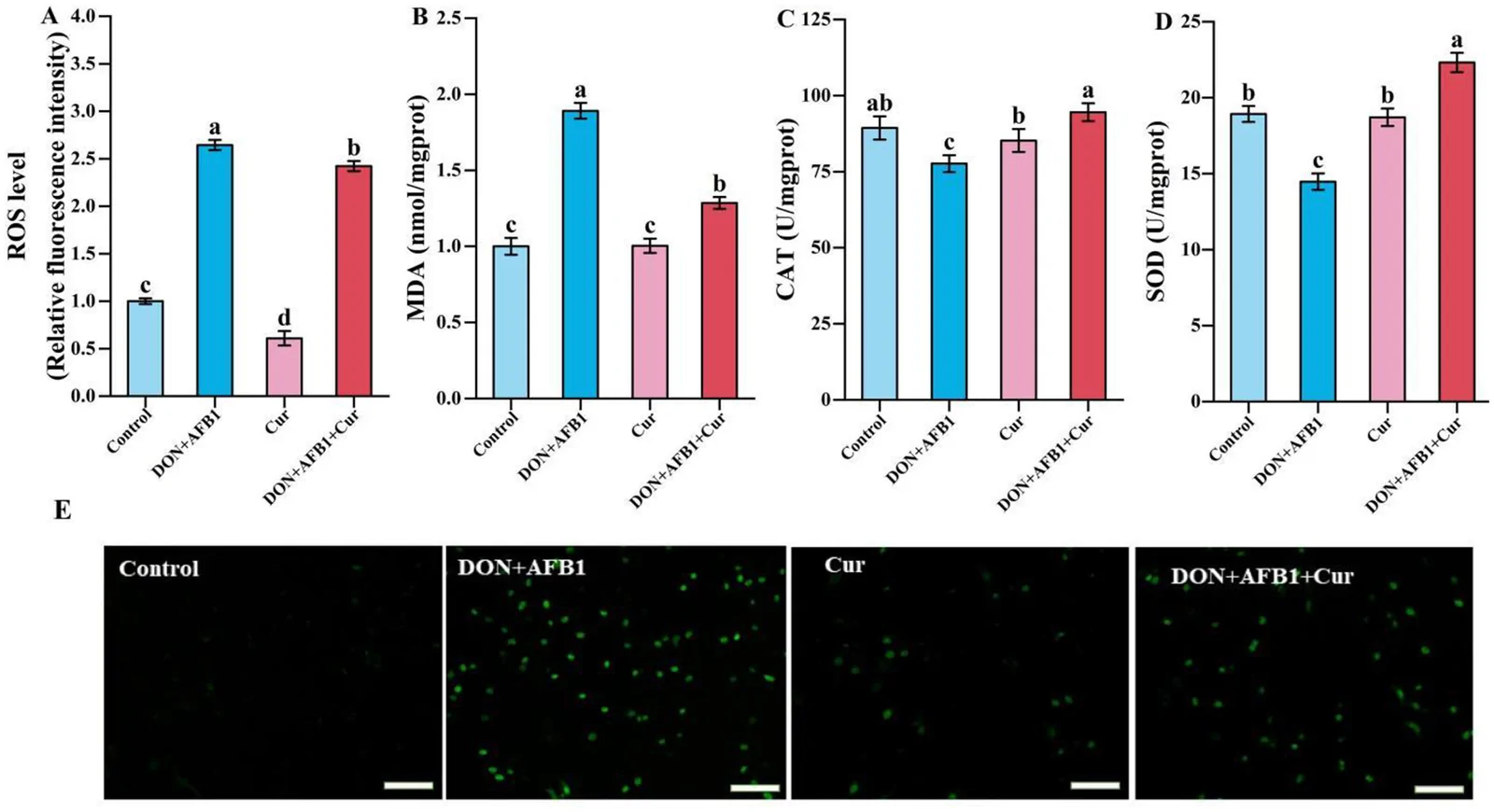
Curcumin attenuates DON+AFB1-induced oxidative stress in PAMs. **(A)** Relative intracellular ROS level. **(B)** MDA content. **(C,D)** CAT and SOD activity. **(E)** Representative confocal images of intracellular ROS visualized with a ROS-sensitive fluorescent dye (10 × magnification). Data are expressed as mean ± SD from three independent experiments. Bars with different lowercase letters indicate statistically significant differences (*P* < 0.05) between groups.

### 3.3 Curcumin increased Nrf2 expression in PAMs

To determine how curcumin protects from DON+AFB1 induced cytotoxicity, expression of the antioxidant pathway components Nrf2, Keap1, HO-1, and NQO1 was examined. DON+AFB1 exposure significantly decreased mRNA levels of Keap1, Nrf2, and HO-1 relative to control, and curcumin pretreatment restored expression of all three (Figures 3A–C). NQO1 mRNA was likewise significantly increased by curcumin pretreatment relative to the DON+AFB1 group (Figure 3D). Protein expression followed the same pattern, Nrf2, HO-1, and NQO1 were reduced by DON+AFB1 and restored by curcumin pretreatment (Figures 3E–H). Together, these results indicate that curcumin protects PAMs against oxidative damage induced by DON and AFB1 in association by upregulation of Nrf2/HO-1 signaling pathway.

**Figure 3:**
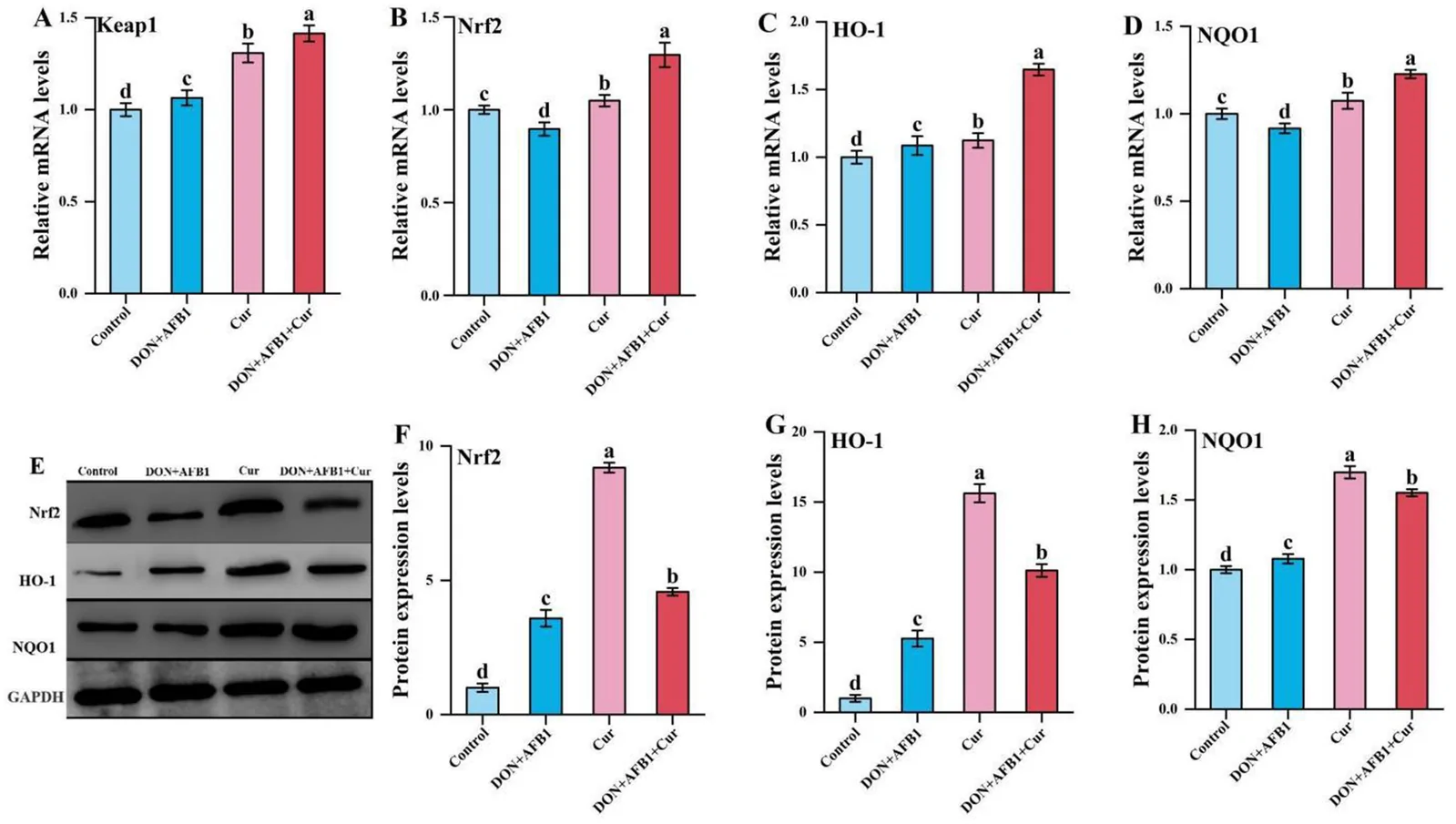
Curcumin restores Nrf2/HO-1 pathway expression suppressed by DON+AFB1 in PAMs. **(A–D)** Relative mRNA levels of Keap1, Nrf2, HO-1, and NQO1. **(E)** Representative western blot images. **(F–H)** Quantification of Nrf2, HO-1, and NQO1 protein expression by ImageJ analysis. Data are expressed as mean ± SD from three independent experiments. Bars with different lowercase letters indicate statistically significant differences (*P* < 0.05) between groups. Total Nrf2 protein levels are shown; functional activation (e.g., nuclear translocation) was not directly assessed.

### 3.4 DON+AFB1-induced apoptosis in PAMs

Co-exposure to DON (0.5 μg/mL) and AFB1 (0.04 μg/mL) for 24 h significantly activated the mitochondrial apoptotic pathway in PAMs. Relative to control, DON+AFB1 co-exposure significantly suppressed protein expression of the anti-apoptotic gene Bcl-2, while significantly increasing expression of the pro-apoptotic markers Bax, caspase-3, and cytochrome c (Figures 4A–D). Curcumin treatment alone maintained baseline expression patterns, and curcumin pretreatment reversed the DON+AFB1-induced changes, elevating Bcl-2 expression while reducing Bax, cytochrome c, and caspase-3 expression. Western blot images (Figure 4E) were consistent with this quantification, confirming that DON+AFB1 promoted apoptosis while curcumin exerted a protective effect. Flow cytometry showed an increased proportion of apoptotic cells following DON+AFB1 co-treatment relative to control (Figures 4F, G); curcumin supplementation markedly reduced this proportion, although it remained slightly above control levels. Together, these findings indicate that curcumin attenuates apoptosis induced by DON and AFB1 in PAMs through regulation of the mitochondrial apoptotic signaling pathway.

**Figure 4:**
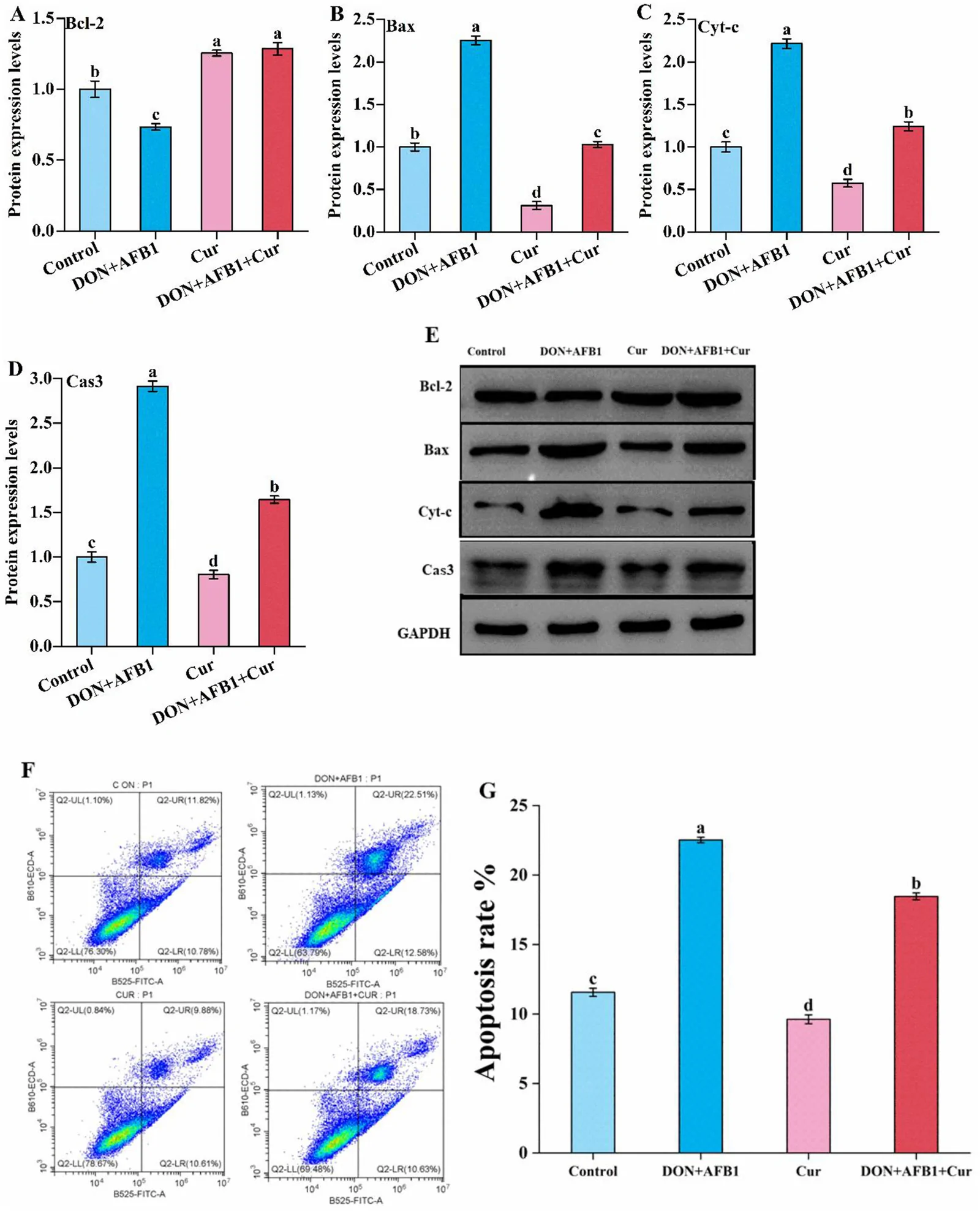
Curcumin attenuates apoptosis induced by DON and AFB1 in PAMs. **(A–D)** Relative protein expression of Bcl-2, Bax, cytochrome c (Cyt-c), and caspase-3 (Cas3), quantified by ImageJ analysis. **(E)** Representative western blot images. **(F)** Flow cytometric analysis of apoptotic cell populations. **(G)** Quantification of apoptosis rate. Data are expressed as mean ± SD from three independent experiments. Bars with different lowercase letters indicate statistically significant differences (*P* < 0.05) between groups.

### 3.5 Effects of ML385 on the protective actions of curcumin in PAMs

The mRNA and protein levels of Bcl-2, Bax, Cyt-c, and caspase-3 were assessed by RT-qPCR and western blot to determine whether Nrf2 inhibition affects the anti-apoptotic effect of curcumin. Relative to control and curcumin-treated groups, DON+AFB1 co-exposure significantly decreased Bcl-2 mRNA (Figure 5A) and increased mRNA levels of caspase-3, cytochrome c, and Bax (Figures 5B–D). Western blot analysis showed the same pattern at the protein level: Bax, cytochrome c, and caspase-3 were elevated in the DON+AFB1 group relative to control, while Bcl-2 was reduced relative to the curcumin-treated group (Figure 5E). Curcumin pretreatment shifted this balance toward cell survival, elevating Bcl-2 and suppressing the three pro-apoptotic markers. Addition of the Nrf2 inhibitor ML385 to curcumin-treated cells altered both mRNA and protein expression of these markers relative to the curcumin-only group, confirming that the anti-apoptotic effect of curcumin in PAMs depends on Nrf2 signaling.

**Figure 5:**
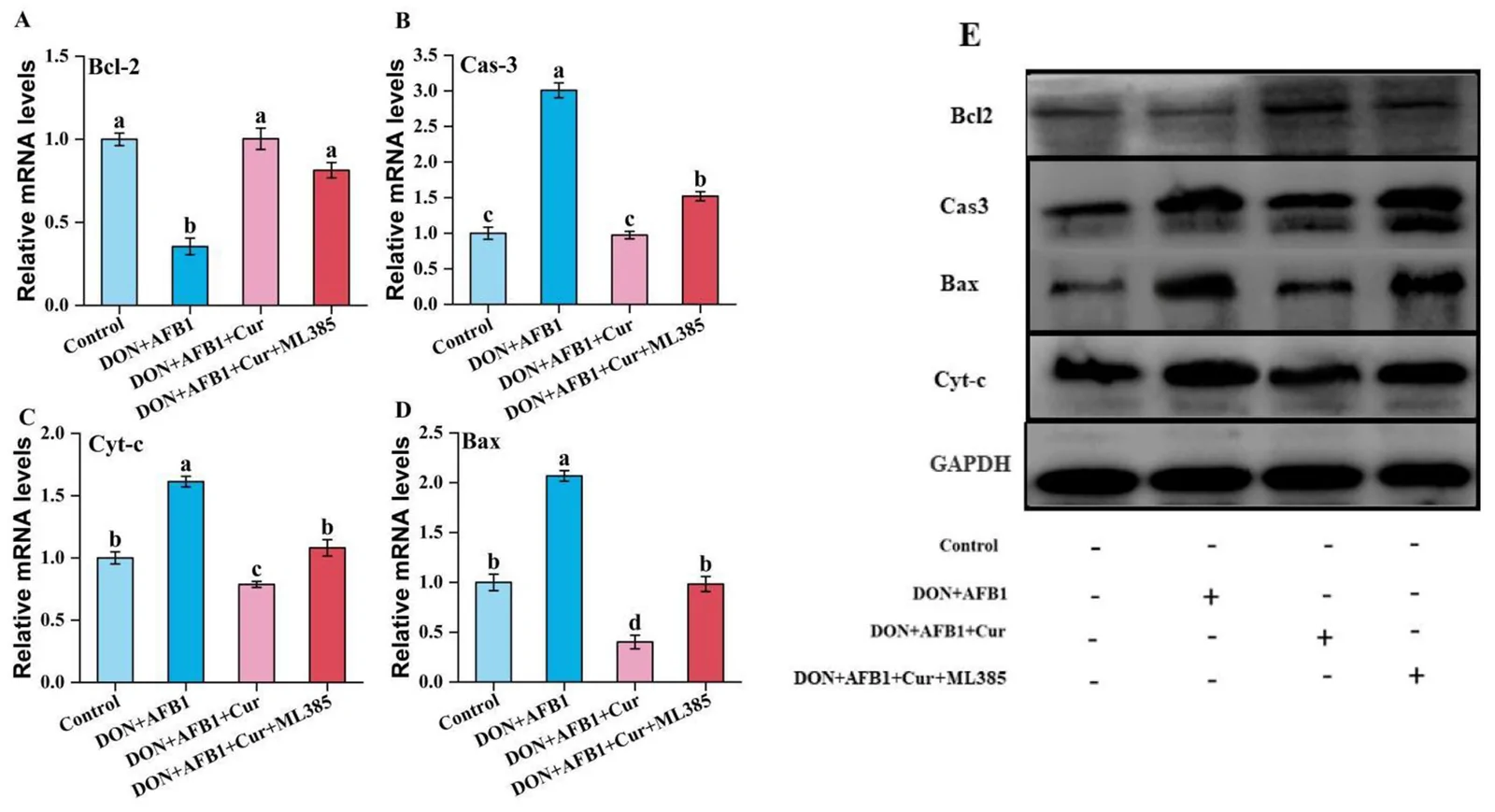
Nrf2 inhibition with ML385 attenuates the anti-apoptotic effect of curcumin in PAMs. **(A–D)** Relative mRNA levels of Bcl-2, caspase-3, cytochrome c (Cyt-c), and Bax, quantified by qRT-PCR. **(E)** Representative western blot images of Bcl-2, caspase-3, Bax, and cytochrome c, with relative protein levels quantified by ImageJ analysis. Data are expressed as mean ± SD from three independent experiments. Bars with different lowercase letters indicate statistically significant differences (*P* < 0.05) between groups.

### 3.6 Effects of ML385 on the Nrf2 pathway in curcumin-treated PAMs

To confirm the involvement of Nrf2 signaling in the protective effect of curcumin, PAM cells were treated with ML385, a selective Nrf2 inhibitor, and transcriptional and protein expression of Keap1, Nrf2, HO-1, and NQO1 were evaluated. Curcumin treatment increased relative mRNA levels of all four targets compared with the DON+AFB1 group. Addition of ML385 significantly decreased mRNA levels of Keap1, Nrf2, HO-1, and NQO1 relative to the curcumin-treated group (Figures 6A–D). Similarly, ML385 addition downregulated Nrf2, HO-1, and NQO1 protein expression relative to the curcumin-treated group, although levels remained higher than in the DON+AFB1-only group (Figure 6E). These results indicate that curcumin activates the Nrf2 pathway in response to toxin-induced ROS, resulting in reduced apoptosis and oxidative stress, and that ML385 reverses this protective effect by inhibiting Nrf2 signaling.

**Figure 6:**
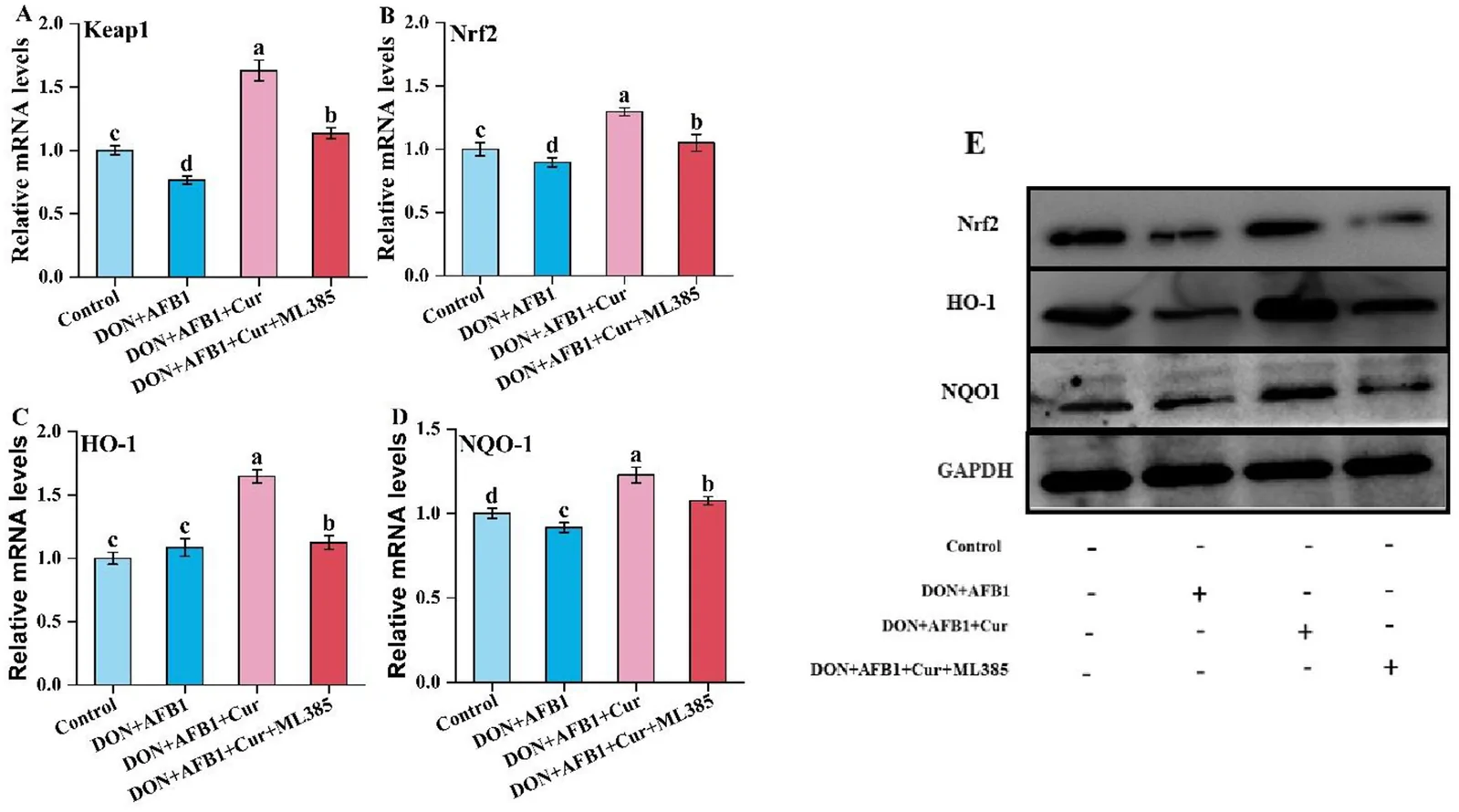
Curcumin upregulates the Nrf2 pathway, an effect attenuated by ML385. **(A–D)** Relative mRNA levels of Keap1, Nrf2, HO-1, and NQO1, quantified by qRT-PCR. **(E)** Representative western blot images of Nrf2, HO-1, and NQO1, with relative protein levels quantified by ImageJ analysis. Data are expressed as mean ± SD from three independent experiments. Bars with different lowercase letters indicate statistically significant differences (*P* < 0.05) between groups.

## 4 Discussion

In this study, integrated data from research demonstrates that combined DON and AFB1 exposure induces oxidative stress and intrinsic apoptotic changes in porcine alveolar macrophages (3D4/21), and that curcumin attenuates these effects in association with activation of the Nrf2/HO-1 pathway. In addition to confirming the general pattern of DON+AFB1 toxicity previously reported by our group and others in systemic and hepatic models (25, 26), the present work makes two contributions. First, it characterizes the effects of DON+AFB1 co-occurrence specifically in a PAM (3D4/21) cell type. Secondly, it examines the involvement of Nrf2 signaling using pharmacological inhibition with ML385.

The working concentrations (DON 0.5 μg/mL; AFB1 0.04 μg/mL) correspond to combined no-observed-adverse-effect levels previously established by this laboratory in 3D4/21 cells and to murine oral no-effect doses of 0.1 mg DON/kg and 0.01 mg AFB1/kg body weight (27). In the viability titration (Figure 1A), this pair was the lowest combination that producing a statistically significant reduction cell viability and was therefore suitable for evaluation of a protective intervention.

Co-contamination of feed with AFB1 and DON is common. A global survey of 74,821 samples from 100 countries detected AFB1 in 23% and DON in 64% of samples, with 64% of all samples containing multiple mycotoxins (28). Among 827 complete pig feed samples from China, AFB1 was detected in more than 83% and DON in more than 74% of samples, with co-occurrence in 96.4%−100% of contaminated samples (29). EU monitoring has reported AFB1 at 0.003–0.042 mg/kg and DON at 0.16–0.95 mg/kg in naturally contaminated feed, under current EU rules, expressed relative to a feeding stuff with 12% moisture content, the binding maximum for AFB1 is 5 μg/kg in compound feed for piglets and 20 μg/kg in compound feed for other pigs (*Directive 2002/32/EC*); the guidance value for DON in pig compound feed is 0.9 mg/kg (*Recommendation 2006/576/EC*) (7).

In pigs, AFB1 undergoes extensive hepatic first-pass oxidation by CYP3A/CYP1A isoforms to the 8,9-epoxide, so circulating parent AFB1 remains low after oral exposure (30). DON is absorbed rapidly in the proximal small intestine, before incomplete microbial de-epoxidation to DOM-1 in the distal gut (31). Respirable feed dust bypasses this sequence and can deliver parent AFB1 and DON directly to alveolar macrophages. AFB1 is enriched on 5–7 μm particles that deposit in the alveolar region, and personal air samples collected during grain handling have contained DON and AFB1 together (32, 33). Experimental intranasal mycotoxin exposure has also been shown to distribute toxin to the liver, kidney and spleen while recruiting alveolar macrophages and neutrophils at the site of deposition (12). Urinary biomarkers confirm absorption of both toxins from piglet diets near current EU guidance values, and comparable biomarkers are detected in humans with dietary exposure, confirming that DON and AFB1 are not merely present in feed but are absorbed and circulating in the bodies of both target livestock species and humans (34, 35). However, these feed and urine values cannot be directly equated with 0.5 and 0.04 μg/mL concentrations used in medium. That conversion would require a validated inhalation physiologically based toxicokinetic model for swine, which is not available.

A 60 kg growing pig, assuming a blood volume of 70 mL/kg (4.2 L) and a daily intake of 2.17 kg complete feed contaminated at the EU levels cited above, would ingest approximately 43.4 μg AFB1 and 1.95 mg DON per day (equivalent to approximately 0.72 and 32.6 μg/kg body weight, respectively). To provide a purely illustrative order of magnitude context, not as a validated toxicokinetic prediction. If one assumes hypothetically that 20% of ingested AFB1 remained as parent compound within that blood volume, a simplification that ignores extensive hepatic first pass extraction, protein binding, tissue distribution, clearance, and that 80% of ingested DON was absorbed into the same volume (a bounding assumption that ignores tissue distribution and clearance), the resulting theoretical maximum concentrations would be approximately 2 ng/mL AFB1 and 370 ng/mL DON. In practice, measured porcine plasma concentrations are typically lower than these bounding estimates. The working concentrations used here (0.5 μg/mL DON and 0.04 μg/mL AFB1) therefore exceed realistic EU derived plasma levels of parent toxin, particularly for AFB1, and were not selected as plasma equivalents; rather they represent an *in vitro* challenge dose grounded in previously established dose response data for this cell line.

These concentrations remain a defensible macrophage challenge when feed provisions outside the European Union are considered. In the United States, FDA action levels permit 200 μg/kg aflatoxin in corn or peanut products intended for finishing swine of at least 100 lb and 100 μg/kg for breeding swine (20 μg/kg for immature animals). Advisory levels for DON in grain for swine are 5 mg/kg, when grain does not exceed 20% of the diet, equivalent to approximately 1 mg/kg in complete feed. In Canada, aflatoxins are limited to 20 μg/kg in the total diet, while DON may reach 1 mg/kg in starter diets and 2 mg/kg in diets for other swine classes. In many grain producing regions, pig specific limits for DON are absent or weakly enforced, and surveys of complete pig feed frequently report DON above 1 mg/kg and AFB1 above 20 μg/kg. The present *in vitro* concentrations therefore constitute a biologically relevant macrophage challenge across this wider exposure range, rather than a claim that European plasma contains 0.5 or 0.04 μg/mL.

Co-exposure to DON+AFB1 reduced PAMs viability and increased LDH release in a dose dependent manner, consistent with previously reported combine mycotoxins induce membrane and cytotoxicity in 3D4/21 and PK-15 cell lines (36, 37). Curcumin pretreatment effectively restored cell viability and reduced LDH leakage in line with earlier reports, that curcumin protects cells against mycotoxin induced cytotoxicity through its antioxidant and membrane stabilizing properties (38). Consistent with the central role of oxidative stress in mycotoxin induced toxicity (39–41), DON+AFB1 markedly increased ROS and MDA while suppressing SOD and CAT activity, indicating both excess ROS generation and impaired antioxidant defense. These results indicate that DON+AFB1 leads to redox imbalance and induces oxidative stress, consistent with previously reported zearalenone promoted ROS production that subsequently triggered apoptosis (42), and are consistent with DON and AFB1 induced ROS production and lipid peroxidation in immune as well as epithelial cells (43). Curcumin pretreatments reversed these changes, restored cellular redox homeostasis, an effect mainly attributable to its phenolic antioxidant structure (44, 45).

Sustained oxidative stress leads to activation of the intrinsic apoptotic pathway. Excessive ROS promotes the mitochondrial outer membrane permeabilization, triggering cytochrome c release into the cytosol and activation of the caspase cascade (46). In the present study, DON+AFB1 co-exposure significantly downregulated Bcl-2 while upregulating Bax, cytochrome c, and caspase-3 at both mRNA and protein levels. Flow cytometry confirmed a corresponding increase in the proportion of apoptotic cells. This pattern is consistent with previous reports that DON and AFB1 co-exposure activates the intrinsic mitochondrial apoptotic pathway in IPEC-J2 and C57BL/6 cells via a similar Bax/cytochrome/caspase route (47). Curcumin pretreatment reversed each of these changes by restoring Bcl-2 expression while suppressing Bax, Cytochrome c, and caspase-3. The relative apoptotic cell fraction was reduced but not fully restored to control levels, these results are consistent with previous findings that curcumin has a protective effect on mitochondrial membrane integrity in SH-SY5Y cells against copper induced neurotoxicity (48). Notably, when the Nrf2 inhibitor ML385 was added to curcumin treated cells, the protective shifts in Bcl-2, Bax, cytochrome c and caspase-3 were attenuated, returning the profile toward that observed with DON+AFB1 alone. These findings indicate that the anti-apoptotic effect of curcumin is associated with Nrf2 activity under the conditions tested.

DON+AFB1 suppressed Nrf2, HO-1 and NQO1 at both the mRNA and protein levels, indicating impaired antioxidant defense (49). Curcumin pretreatment markedly restored the expression of these antioxidant genes and proteins. Importantly, pharmacological inhibition of Nrf2 with ML385 abolished this protection, reversing the curcumin mediated restoration of Nrf2/HO-1/NQO1 and increasing Bax, cytochrome c and caspase-3 while reducing Bcl-2. These findings support a role for Nrf2 signaling in the protective effects of curcumin against combined DON+AFB1 induced oxidative and apoptotic injury. These findings build on previous correlative evidence implicating Nrf2 in mycotoxin protection by demonstrating an association between Nrf2 signaling and curcumin mediated protection in porcine alveolar macrophages exposed to combined DON and AFB1 (50).

The present study has several limitations. All experiments were performed in a single porcine alveolar macrophage cell line (3D4/21), without validation in primary PAMs or a parallel epithelial model, which limits conclusions about cell type specificity. The acute *in vitro* exposure does not reflect the chronic, low level dietary exposure or mycotoxin deposition from farm dust. Inhaled dose, plasma concentration, and lung tissue concentration of AFB1 and DON were not measured. Therefore, working concentrations cannot be taken as equivalents of porcine blood or alveolar lining fluid. Additionally, only one pair of toxin concentrations and a single curcumin pretreatment were tested in depth, and Nrf2 involvement was examined by pharmacological inhibition with ML385. Thus, these findings do not establish *in vivo* respiratory protection, occupational risk, or a field level efficacy. Studies using primary porcine cells, physiologically relevant exposure models and a broader concentration range are warranted to further validate these observations.

## 5 Conclusions

In summary, we demonstrated that combined DON and AFB1 exposure can induce oxidative stress and intrinsic apoptosis in a porcine alveolar macrophage line (3D4/21). Curcumin can attenuate these effects in association with Nrf2/HO-1 signaling. Pharmacological inhibition with ML385 indicates that this attenuation is associated with Nrf2 activity under the conditions tested. These findings are preliminary and confined to an acute *in vitro model*; they do not demonstrate *in vivo* or occupational protection and support only further experimental evaluation of curcumin and Nrf2 in this cell type.

## Data Availability

The datasets presented in this study can be found in online repositories. The names of the repository/repositories and accession number(s) can be found in the article/supplementary material.
